# New Biomaterials and Regenerative Medicine Strategies in Periodontology, Oral Surgery, Esthetic and Implant Dentistry 2018

**DOI:** 10.1155/2019/1363581

**Published:** 2019-12-04

**Authors:** David M. Dohan Ehrenfest, Adriano Piattelli, Gilberto Sammartino, Hom-Lay Wang

**Affiliations:** ^1^Department of Oral and Maxillofacial Surgery, The University of Michigan Health System, Ann Arbor, Michigan, USA; ^2^Department of Periodontics and Oral Medicine, The University of Michigan, School of Dentistry, Ann Arbor, Michigan, USA; ^3^LoB5 Research Unit, School of Dentistry & Research Center for Biomineralization Disorders, Chonnam National University, Gwangju, Republic of Korea; ^4^Department of Medical, Oral and Biotechnological Sciences, G. D'Annunzio University of Chieti-Pescara, Chieti, Italy; ^5^Department of Oral Surgery, Faculty of Medicine, University of Naples Federico II, Naples, Italy

The development of new biomaterials and regenerative medicine strategies appears as a major field of research in periodontology, oral surgery, and esthetic and implant dentistry (the POSEID disciplines) in the current era of tissue engineering and quest for tissue regeneration. Biomaterial research remains the core science of all these opportunities of regenerative treatments of the maxilla and improvement of oral rehabilitation [1].

In our previous special issues in 2015 and 2016, we selected a series of articles with new data on a wide range of topics in biomaterials and regenerative research in periodontology, oral surgery, and esthetic and implant dentistry, and we highlighted how these interconnected fields of research are both transversal (multidisciplinary) and translational (from basic sciences to clinical applications). In this 2018 special issue, we selected articles with the same insight and wished to illustrate the complexity of this research field.

In our previous editorials in 2015 and 2016, we described particularly the strength, weakness, opportunities, and threats on these disciplines and how research in implantable materials, particularly dental implants (new implant design and surfaces) [2], bone materials, or surgical adjuvants [3], is affected by not only scientific bias and misunderstandings, but also industrial and financial interferences, creating many inaccuracies in the literature. Despite the incredible potential and revolution these disciplines are supporting for patients and the clinical approach of oral rehabilitation, there is also a major threat to the credibility of this field.

In the 2016 editorial of this special issue, we described particularly some ethical and legal issues related to this field, especially concerning the lack of transparency and control in dental implants (with so many illegal pirate productions or the lack of quality controls in general) or through the dramatic example of L-PRF (leukocyte- and platelet-rich fibrin) and related blood derivatives (with many providers marketing kits and devices for blood concentrates without any legal authorization in many countries, for example, A-PRF and i-PRF) [4]. These major issues were creating confusion in the mind of users, but also in the literature some researchers received undisclosed funding to write articles about techniques or materials [5], which sometimes were marketed but not even legally authorized! This was one of the major threats to this research field: how the credibility of some research topics was affected by the lack of ethics of some authors and merchants [5].

In 2018, unfortunately, very little has evolved. As a strange matter of fact, the development of these new biomaterials and regenerative medicine strategies seems to have reached a plateau that no one seemed to expect so early, before any major breakthrough or revolution of the clinical paradigms. As we can observe in the current literature, research topics seem to be permanently turning around, and we see currently very little major developments.

In this 2018 special issue on new biomaterials and regenerative medicine strategies in periodontology, oral surgery, and esthetic and implant dentistry, we continued our task to gather a meaningful corpus of relevant articles. More than before, a better control of the specialized literature is needed.
